# Synaptic Effect of Aδ-Fibers by Pulse-Train Electrical Stimulation

**DOI:** 10.3389/fnins.2021.643448

**Published:** 2021-04-26

**Authors:** Shota Tanaka, Jose Gomez-Tames, Koji Inui, Shoogo Ueno, Akimasa Hirata, Toshiaki Wasaka

**Affiliations:** ^1^Department of Electrical and Mechanical Engineering, Nagoya Institute of Technology, Nagoya, Japan; ^2^Center of Biomedical Physics and Information Technology, Nagoya Institute of Technology, Nagoya, Japan; ^3^Department of Functioning and Disability, Aichi Developmental Disability Center, Institute for Developmental Research, Kasugai, Japan; ^4^Department of Integrative Physiology, National Institute for Physiological Sciences, Okazaki, Japan; ^5^Department of Biomedical Engineering, Graduate School of Medicine, The University of Tokyo, Tokyo, Japan; ^6^Frontier Research Institute for Information Science, Nagoya Institute of Technology, Nagoya, Japan

**Keywords:** intraepidermal electrical stimulation, small fibers, Aδ-fiber, perception/pain, electromagnetic model, nerve model, synaptic model, multiscale model

## Abstract

Electrical stimulation of specific small fibers (Aδ- and C-fibers) is used in basic studies on nociception and neuropathic pain and to diagnose neuropathies. For selective stimulation of small fibers, the optimal stimulation waveform parameters are an important aspect together with the study of electrode design. However, determining an optimal stimulation condition is challenging, as it requires the characterization of the response of the small fibers to electrical stimulation. The perception thresholds are generally characterized using single-pulse stimulation based on the strength-duration curve. However, this does not account for the temporal effects of the different waveforms used in practical applications. In this study, we designed an experiment to characterize the effects of multiple pulse stimulation and proposed a computational model that considers electrostimulation of fibers and synaptic effects in a multiscale model. The measurements of perception thresholds showed that the pulse dependency of the threshold was an exponential decay with a maximum reduction of 55%. In addition, the frequency dependence of the threshold showed a U-shaped response with a reduction of 25% at 30 Hz. Moreover, the computational model explained the synaptic effects, which were also confirmed by evoked potential recordings. This study further characterized the activation of small fibers and clarified the synaptic effects, demonstrating the importance of waveform selection.

## Introduction

The central nervous system receives somatosensory information from different receptors and peripheral nerve fibers, which are integrated by synaptic processes. Free nerve endings or terminals of small fibers, such as the Aδ- and C-fibers, are located in the epidermis. Selective stimulation is essential for investigating somatosensory submodality and pain processes (Vallbo et al., 1979). Intraepidermal electrical stimulation (IES) using a small concentric bipolar needle electrode that injects a current of a few mA to generate a focal electric field around the electrodes can selectively stimulate small fibers (Inui and Kakigi, 2012). Different stimulation parameters (for example, duration, interstimulus interval, waveform, and electrode polarity) have been proposed to facilitate the selective stimulation of Aδ- and C-fibers. Stimulation of different types of small fibers can be confirmed by measuring the reaction times and recording pain-related evoked potentials (Inui and Kakigi, 2012; Kodaira et al., 2014; Hugosdottir et al., 2019). However, defining the optimal stimulation conditions for these small fibers is challenging as they depend on the design of the electrode, characteristics of the stimulated fibers, and how the information is integrated into the synaptic process on the spinal cord and cognitive levels (Inui et al., 2005; Otsuru et al., 2009).

The first approach to characterize the stimulation of small fibers is the strength-duration relationship approach (S-D curve), which shows the threshold relationship between pulse amplitude and duration. The electrostimulation threshold becomes small with an increase in pulse duration until its convergence to a minimum value termed “rheobase.” Two studies measured the perception threshold to derive S-D curves but were not specific to a particular small fiber type (co-activation of different small fibers) (Hennings et al., 2017; Poulsen et al., 2020). Electromagnetic dosimetry has also been used to evaluate the internal electric field as a metric of stimulation in the skin region where the fibers are located (Mørch et al., 2011; Frahm et al., 2013; Hirata et al., 2013a; Motogi et al., 2016).

The effects of the computed internal electric field on the small fiber models were considered. Poulsen et al. (2020) used a fiber model to estimate the activation threshold with matching S-D curve measurements for a limited number of pulse durations. For simplicity, the computed thresholds of a single fiber model were assumed to be equal to the perception threshold based on *in vivo* experiments. In our previous study (Tanaka et al., 2021), the Aδ-fiber model was characterized based on S-D measurements considering the region where multiple fibers could be stimulated. These multiscale models (skin volume conductor and neural models) described local responses at fiber terminals and helped us to better understand the mechanism of IES for single-pulse stimulation. However, they were not sufficient to describe the perception thresholds for non-single-pulse stimulation, wherein the synaptic process became relevant.

Synaptic models incorporate afferent information in the form of temporal/spatial summation to describe variations in the postsynaptic membrane potential (Roth and van Rossum, 2013; Heshmat et al., 2020). If the membrane potential reaches a threshold, the postsynaptic neuron is fired. Experiments on the primary motor have shown that the motor threshold changes with the stimulation waveform (Lilly et al., 1952; Taniguchi et al., 1993), and its effects have been replicated by adopting multiscale modeling with a conductance-based synaptic model (Gomez-Tames et al., 2018, 2019). These complex factors need to be further characterized for peripheral stimulation to optimize protocols for selective stimulation of small fibers.

The present study aimed to measure and characterize the effects of stimulation of a train of pulses on the perception threshold via stimulation of Aδ-fibers *in vivo* for the first time. Stimulation of Aδ-fibers and synaptic effects were confirmed by the reaction time and evoked potential measurements. In addition, we present a synaptic model that integrates the responses of the Aδ-fiber information via a comparison with experiments. One of the features of this study is that the computational model combines electromagnetic dosimetry and nerve activation modeling to integrate the propagation pulses descending from the fiber terminal into a synaptic model.

## Model and Methods

### The IES Experiment

We measured the perception threshold for pulse-train electrical current stimulation with a variable number of pulses (with a fixed frequency) and frequencies (with a fixed number of pulses). The number of healthy participants was eight (age 20.8 ± 1.2 years, five females) and nine (age 21.6 ± 1.3 years, two females) for pulse number and frequency variation, respectively. The experiments were approved by the Ethical Committee of the Nagoya Institute of Technology (no. 29–014).

**FIGURE 1:**
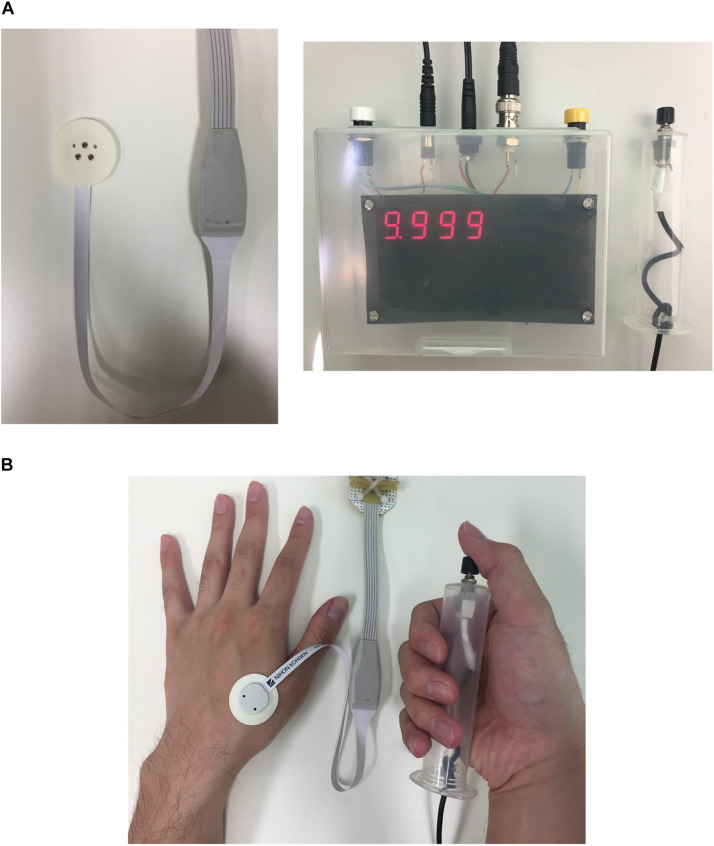
Experimental setup: **(A)** concentric bipolar electrode needle and measurement equipment of reaction time (time between stimulation and detection of sensation) and **(B)** photo of a participant pressing the push-button during perception threshold detection experiment.

The stimulation device (STG4004, Multi-Channel Systems GmbH, Germany) delivered multiple square pulse currents through a concentric bipolar needle electrode (NM-983 W, Nihon Kohden, Tokyo, Japan). The inner needle and ring electrodes were assigned as the anode and cathode of the stimulation device, respectively. The stimulation was applied to the dorsum of the left hand to stimulate Aδ-fibers, as shown in Figure 1. The number of pulses varied from 1 to 10 (one pulse step) using a frequency of 30 Hz. We then evaluated the frequency from 10 Hz to 200 Hz (nine frequencies) using six pulses. The conditions were selected randomly for the experiments.

The experimental protocol to measure the perception threshold (the lowest current to generate sensation in participants) was presented in our previous study (Tanaka et al., 2021). In brief, the participants were instructed to press a button as soon as they felt the sensation. The time between stimulus onset and detection, by pressing a button (termed reaction time), was automatically recorded. The method of limits was used to determine the threshold of the stimulus with consecutive ascending and descending trials. The threshold was considered to be detected when the participant reported a sensation at least two of three times with reaction time in the range of Aδ-fiber transmission (200 to 800 ms) (Ragé et al., 2010; Kodaira et al., 2014).

### Pain-Related Evoked Potentials by Aδ-Fiber

In order to confirm the activation of Aδ-fibers and synaptic effect, we measured the pain-related evoked potentials of Aδ-fibers by electroencephalography (EEG) recordings in three healthy participants (age, 23.3 ± years, all-males) and two healthy participants (age, 22.5 ± 1.5 years, all-males), respectively. First, we confirmed the pain-related evoked potentials of Aδ-fibers for single-pulse stimulation using different pulse widths (60, 100, 200, 400, 800, and 1600 μs). Second, we demonstrated the synaptic effect by recording pain-related evoked potentials for pulse-train electrical stimulation with a different number of pulses using a fixed stimulus injection current. The selected injection current amplitude corresponded to the perception threshold for eight consecutive pulses. We then investigated the presence or absence of pain-related evoked potentials when fewer pulses were applied (two, four, and six).

The protocol for EEG recording following each stimulus condition was described. The active electrode was placed at the Cz of the International 10–20 system and referred to as the linked earlobes (A1-A2). We focused on the cortical response recorded from the Cz since a previous study showed that the maximum response following noxious stimuli was recorded from the Cz (Kakigi et al., 1989). The impedance of the electrode was less than 5 kΩ. In addition, a pair of electrodes placed supra- and infra-orbitally to the left eye was used to record the electrooculogram (EOG). The EEG signals were recorded with a band-pass filter (0.5 to 30 Hz) at a sampling rate of 1000 Hz. The EEG signals were then averaged from the 20 stimuli applied in each pulse condition. Epochs in which the signal variations were larger than 80 μV in the EEG and EOG were excluded. Thereafter, a total of 10–15 stimuli were averaged for each condition. The analysis was conducted from 100 ms before to 800 ms after the onset of the IES. We used the 100 ms period before stimulation as the baseline. For the peak determination of the waveform, we adopted a threshold of more than three times the standard deviation calculated from the prestimulus period. During the EEG recording, we instructed the participant not to pay attention to noxious stimuli in order to eliminate the attentional effect. After the EEG recordings, we asked the participants if they felt any noxious sensation. The participants reported that they felt slight pain with 6- and 8-pulse stimulation, but no pain with fewer pulses.

### Multiscale Modeling of Aδ-Fibers

A computational multiscale electromagnetic model of Aδ-fibers during IES was developed in our previous studies (Motogi et al., 2016; Tanaka et al., 2021). This was based on strength-duration measurements using single-pulse stimulation. A summary of the results is provided in this section. In section “Synaptic Model,” a model of the synapse is incorporated to describe the pulse-train stimulation of small fibers for the first time.

#### Skin Volume Conductor Modeling

**FIGURE 2:**
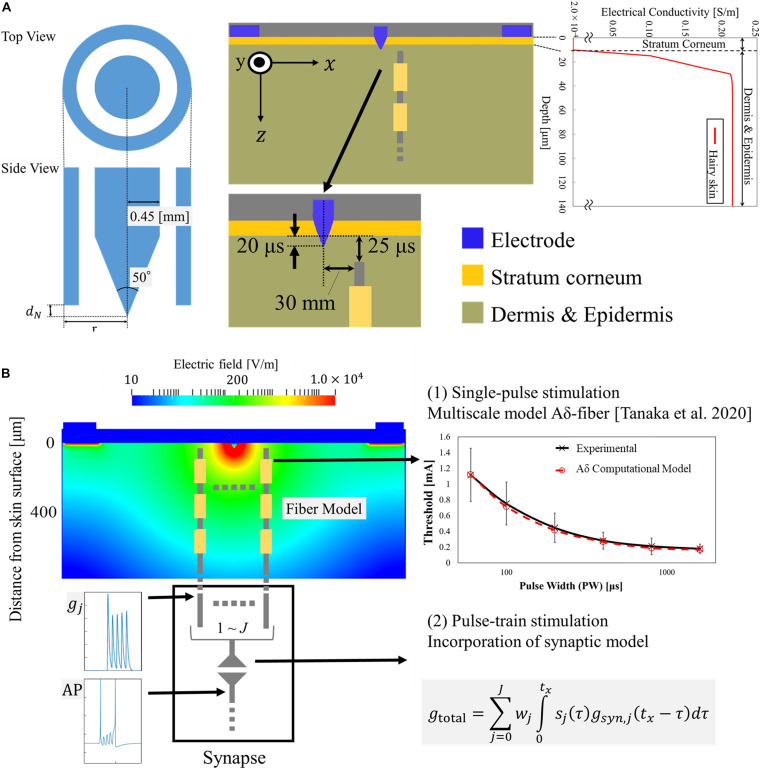
Multiscale electrical stimulation model with synaptic effect: **(A)** Layered skin model with Aδ-fiber and IES electrode model. Profile of electrical conductivity estimated from the water content of the tissue. **(B)** The electric field distribution on the transversal plane of the skin model is illustrated, as an example, for an injection current of 0.02 mA. The response of the Aδ-fiber matched the experimental strength-duration curves for single-pulse stimulation in our previous study (Tanaka et al., 2021). In this work, the afferent spikes of the Aδ-fiber originated from the pulse-train are computed and integrated into a synaptic model.

The electrical skin model is treated as a passive volume conductor to compute the *in situ* electric field produced by the injection current. The skin was modeled as a layered structure for hairy skin (Alekseev and Ziskin, 2007; Schmid et al., 2013; De Santis et al., 2015; Motogi et al., 2016). The thickness of the tissues and their conductivity values were identical to those observed in our previous studies and are summarized in Figure 2A (Tanaka et al., 2021). The dimensions of the skin model were 1.54 mm (depth) × 1.65 mm × 1.65 mm discretized by voxels of 5 μm in length.

The scalar potential finite difference method (Dawson and Stuchly, 1996) was used to numerically solve the following equation to obtain the scalar potential ϕ considering that the frequency is below the kHz range and displacement current is negligible (Hirata et al., 2013b):

(1) ∇⁡(σ∇⁡(φ))=0

where σ is the conductivity of the tissue. The potential was solved iteratively using the successive-over-relaxation and multigrid methods (Laakso and Hirata, 2012). A current source was connected between the inner and outer rings of the bipolar needle electrode (Motogi et al., 2014). The inner electrode and ring electrode corresponded to the cathode and anode, respectively, and were modeled as perfect conductors. To obtain the in situ electric fields, the potential difference between the nodes of the voxel was divided by the voxel length.

#### Nerve Activation Modeling for Aδ-Fibers

The effects of the extracellular electric field derived from Eq. (1) on myelinated nerve fibers were described using the following general equation (McNeal, 1976; Rattay, 1999):

(2) $$\begin{array}{c} C_{m,n}\frac{dV_{m,n}}{dt}+I_{ion,n}-\frac{V_{m,n-1}-2V_{m,n}-2V_{m,n+1}}{0.5(R_{m,i+R_{m,n})}}\\ =\frac{V_{e,n-1}-2V_{e,n}-2V_{e,n+1}}{0.5\left(R_{m,i}+R_{m,n}\right)} \end{array}$$

where *C*_*m*_ is the membrane capacitance, and *R_*m,i*_*, and *R*_*m,n*_ are the internode membrane resistivity and nodal membrane resistivity, respectively. The membrane potential is represented by *V*_*m*,_ where *n* = *V*_*e*_ - *V*_*i*_. The fibers are formed by internodes (myelin segments) and nodes of Ranvier (ionic channels). At the internodes, the membrane current *I*_*ion,n*_ was modeled by the passive conductance multiplied by the membrane potential. At the nodes of Ranvier, the ionic membrane current was formulated using a modified Chiu–Ritchie–Rogart–Stagg–Sweeney model, which is a conductance-based voltage-gated model (Sweeney et al., 1987).

The parameters in the modified fiber model were explored in the electromagnetic computation model based on reported values in the literature (depth and diameter) (MacIver and Tanelian, 1993; Reilly et al., 1997) to fit the experimental strength-duration experiment for single-pulse stimulation. The area of stimulation was estimated based on the rheobase value of the strength-duration experiment. The conductance and capacitance of the cable equation were modified to adjust the chronaxie value (Sweeney et al., 1987) using the least-square error for each pulse.

### Synaptic Model

We integrated the responses of the Aδ-fiber model into a synaptic model to investigate the synaptic effects of pulse-train electrical stimulation, as shown in Figure 2B. The generated spikes (namely, action potentials) from Aδ-fibers in the skin produced an excitatory postsynaptic current (EPSC) in the postsynaptic membrane. The EPSC produces a variation in the transmembrane potential of the postsynaptic neuron that can generate an action potential if the membrane potential reaches a minimum threshold. To obtain the EPSC, we first modeled the synaptic conductance as the sum of two exponentials (Roth and van Rossum, 2013):

(3) $$g_{j}=g_{max,j}f\left(e^{-\frac{t}{\tau_{f,i}}}-e^{-\frac{t}{\tau_{r,j}}}\right)$$

where *g*_*maxx,j*_ is the peak conductance, *and* τ_*r,j*_, and τ_*f,j*_ are the rise and fall time constants, respectively. The normalization factor *f* (Roth and van Rossum, 2013) is set such that the amplitude is equal to *g*_*maxx,j*_.

The total synaptic conductance *g*_*total*_ is calculated by combining the effects of each synapse *j* as the convolution of the synaptic conductance *g*_*j*_ at time *t*_*x*_ and the spike sequences *s*_*j*_ arriving from the presynaptic neuron (Aδ-fibers), as follows:

(4) $$g_{total}\left(t_{x}\right)=\sum_{j=0}^{j}w_{j}\int_{0}^{t_{x}}s_{j}\left(\tau \right)g_{j}\left(t_{x}-\tau \right)d\tau$$

where *J* is the total number of presynaptic neurons (Aδ-fibers), and *s*_*j*_ is the delta pulse. The parameter *w*_*j*_ is a weighting term, meaning the probability of depolarizing the synapse before the input and generating action potentials.

Then, the EPSC is given as follows:

(5) $$EPSC\left(t_{x}\right)=g_{total}\left(t_{x}\right)\left[E-V_{m}\left(t_{x}\right)\right]$$

where E is the synaptic reversal potential, and Vm is the postsynaptic membrane potential. We evaluated the synaptic responses in a postsynaptic neuron using an Izhikevich spike model (Izhikevich, 2003), as follows:

(6) $$\frac{dV_{m}}{dt}=0.04V_{m}^{2}+5V_{m}+140-u+EPSC$$

(7) $$\frac{du}{dt}=0.02\left(0.2V_{m}-u\right)$$

Where *u* is the membrane recovery variable.

In this study, we investigated the number of presynaptic inputs (number of individual fibers) required to activate postsynaptic neurons under different train-pulse conditions. We then used the relationship between the current threshold and the number of presynaptic neurons to obtain the injection current threshold to activate the postsynaptic neuron (Supplementary Figure 1), as described in section “Development of a Computational Synaptic Model.” This approach has been used in synaptic models for brain stimulation of the motor area (Gomez-Tames et al., 2019).

## Experimental and Computational Results

### Experiment Results of Aδ-Fibers for Pulse-Train Electrical Stimulation

**FIGURE 3:**
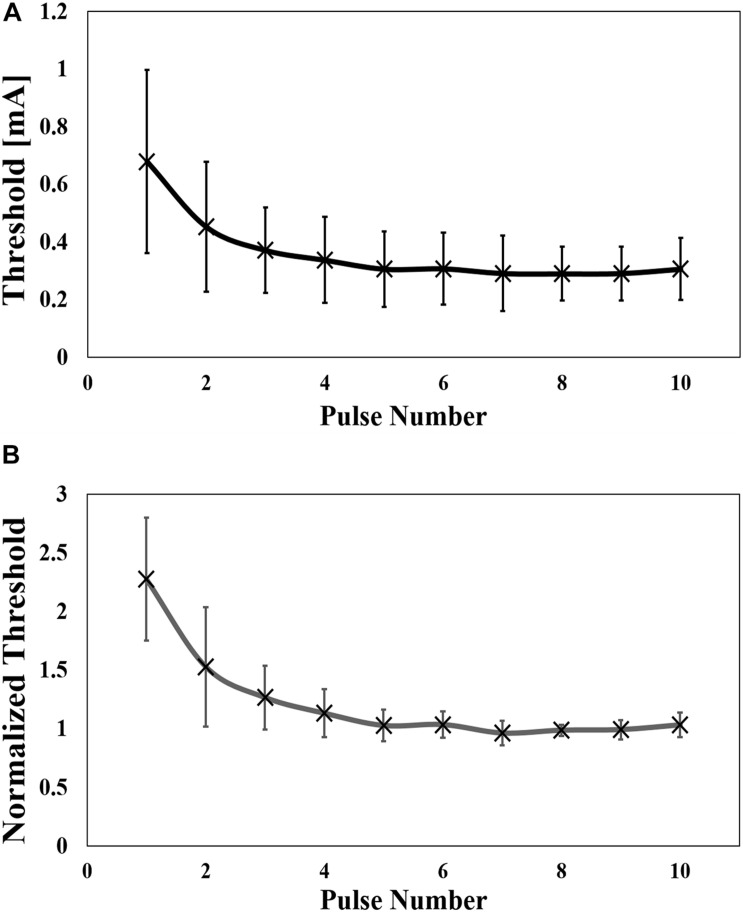
**(A)** Experimental perception threshold variation with pulse number by a pulse-train electrical stimulation (mean value and standard deviation, *n* = 8), and **(B)** its normalization by the average of the thresholds from six to ten pulses (mean value and standard deviation).

The injection current threshold to generate pain perception via Aδ-fibers was experimentally obtained for pulse-train electrical stimulation at a fixed frequency (30 Hz). As shown in Figure 3A, the threshold decreased with an increase in the pulse number and converged after five consecutive pulses. The synaptic effect generated a reduction of the threshold by 2.3 times for multiple-pulse stimulation of five or more consecutive pulses to a single pulse. Higher variability was observed for a few pulses due to intrinsic skin morphology and needle depth variability in the experiments, which will be discussed below. Thus, the measured perception thresholds were normalized by the average of the thresholds from six to ten pulses (Figure 3B). The reaction time of the volunteers in Table 1 requires more time for a larger number of pulses.

**TABLE 1:** The reaction time of perception threshold at a different number of pulses and frequencies.

Pulse number	Reaction time [s] (Mean ± SD)	Frequency [Hz]	Reaction time [s] (Mean ± SD)
1	0.521 ± 0.112	10	0.642 ± 0.060
2	0.487 ± 0.073	20	0.586 ± 0.087
3	0.496 ± 0.088	30	0.508 ± 0.078
4	0.559 ± 0.126	40	0.508 ± 0.041
5	0.606 ± 0.124	50	0.479 ± 0.054
6	0.615 ± 0.110	60	0.490 ± 0.117
7	0.641 ± 0.059	80	0.484 ± 0.098
8	0.631 ± 0.069	100	0.427 ± 0.083
9	0.609 ± 0.063	200	0.422 ± 0.094
10	0.641 ± 0.113		

*SD, standard deviation.*

**FIGURE 4:**
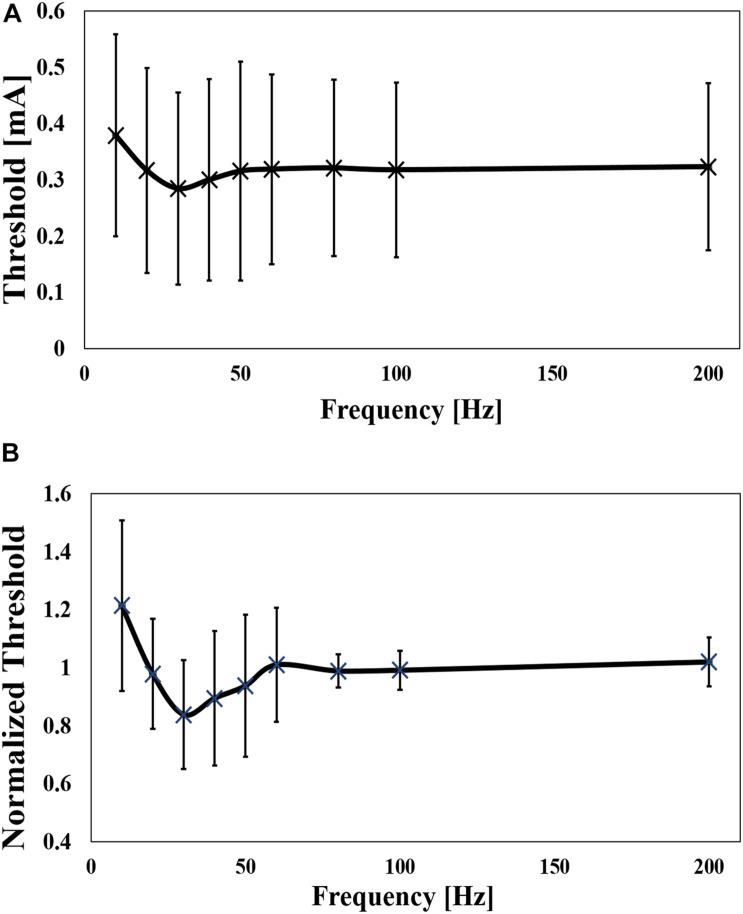
**(A)** Experimental perception threshold variation with frequency by a pulse-train electrical stimulation (mean value and standard deviation, *n* = 9), and **(B)** its normalization by the average of the thresholds between 80 Hz and 200 Hz (mean value and standard deviation) (Mean value and standard deviation).

The dependence of the threshold was also investigated for frequency (10 Hz to 200 Hz), as shown in Figure 4A for six pulses. The mean value of the minimum threshold was found at 30 Hz (10 Hz and 80 Hz), which converged at a mean frequency of 120 Hz (80 Hz to 200 Hz). The threshold was reduced by up to 26% of the maximum value. To reduce the intrinsic variability in the experiments, the threshold was normalized by the mean value of the converged threshold, which was defined as the threshold at a frequency of 80 Hz to 200 Hz (Figure 4B). The normalized curve shows a clear tendency, confirming the bottom peak at 30 Hz. As shown in Table 1, the reaction time was slower at lower frequencies.

### Verification of Aδ-Fibers and Synaptic Effect by Evoked Potentials

We detected evoked potentials from Aδ-fiber stimulation using single-pulse stimulation. Table 2 shows the peak latency of pain-related evoked potentials that were not significantly affected by pulse width (330–390 ms). These reaction times were faster than those when the participant pressed the button under the same conditions in our previous study (470 ms to 520 ms) (Tanaka et al., 2021).

**TABLE 2:** Experimental threshold and peak latency of pain-related evoked potentials by single-pulse stimulation (mean value and standard deviation, *n* = 3).

Pulse width (μs)	Threshold [mA] (Mean ± SD)	Reaction time by evoked potential latency [s] (Mean ± SD)
60	0.983 ± 0.152	0.363 ± 0.020
100	0.647 ± 0.084	0.364 ± 0.085
200	0.487 ± 0.021	0.330 ± 0.039
400	0.330 ± 0.045	0.370 ± 0.035
800	0.323 ± 0.021	0.380 ± 0.101
1600	0.257 ± 0.049	0.391 ± 0.015

*SD, standard deviation.*

**FIGURE 5:**
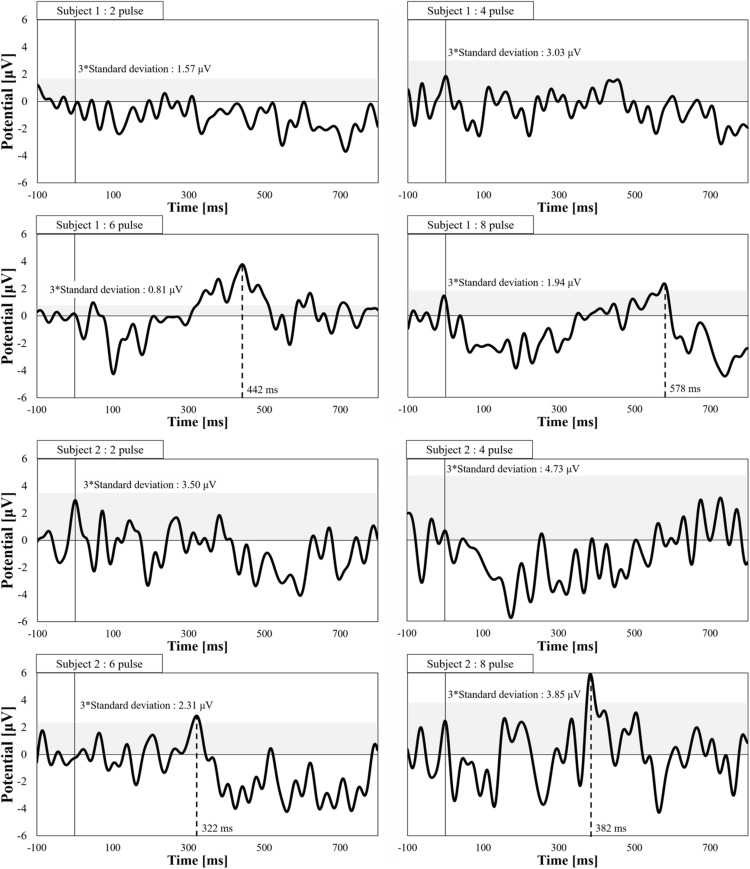
Electroencephalogram waveforms of pain-related evoked potentials by a pulse-train electrical stimulation (frequency of 30 Hz and pulse width of 400 μs, *n* = 2). The peak detection corresponds to values over three times the standard deviation of prestimulus time.

We also verified synaptic effects using evoked potential measurements. Figure 5 shows the evoked potentials for different numbers of consecutive stimulation pulses. The stimulation amplitude was fixed for all conditions (perception threshold of eight pulses). We observed pain-related evoked potentials at eight and six pulses, but not for two or four pulses, which agrees with the higher stimulation intensities for fewer pulses, as observed in Figure 3.

### Development of a Computational Synaptic Model

The electrical parameters of the synaptic model were found to coincide with the experimental data shown in Figure 3. The least-squares error between the experimental and computational results was adopted.

The synaptic model considers the effect of the number of activated presynaptic neurons (estimated computationally by the multiscale model of Aδ-fibers in section “Multiscale Modeling of Aδ-Fibers”) and their afferent spike sequence (number of stimulating pulses). Both inputs were used to determine the activation of postsynaptic neurons using a synaptic model. The parameters of the synaptic model are as follows: synaptic weight (*w*), rise time (τ_*r*_), and fall time (τ_*f*_).

First, more fibers are activated at higher injection current as the region where activation occurred became larger (broader and deeper) from a biophysical perspective. The number of stimulated fibers was estimated using the multiscale Aδ-fiber model for different injection currents, considering a uniform fiber density (Ebenezer et al., 2007). Thereafter, the relationship between the number of fibers and injection current was obtained, as shown in Appendix A. Next, the synaptic weight was determined so that the estimated number of fibers activate the postsynaptic neuron under single-pulse stimulation condition (no synaptic effect condition). Second, the number of fibers (corresponding to the current amplitude of the IES) required to activate the postsynaptic neuron was computed for different numbers of train pulses. The required number of fibers to activate the postsynaptic neuron was used to determine the perception thresholds based on the relationship between the number of fibers and the injection current. Third, the parameters τ_*r*_ and τ_*f*_ were adjusted to fit the experimental thresholds for each number of pulses, as shown in Figure 3. The fitted parameters of the synaptic model are presented in Table 3.

**TABLE 3:** Estimated synaptic model parameters based on the measured results.

Parameter	Value
Number of fibers at a single pulse	71
Rise time constant (τ_*r*_)	4.0 ms
Fall time constant (τ_*f*_)	5.0 ms
Synapse weight (*w*)	8.1 × 10^–3^

**FIGURE 6:**
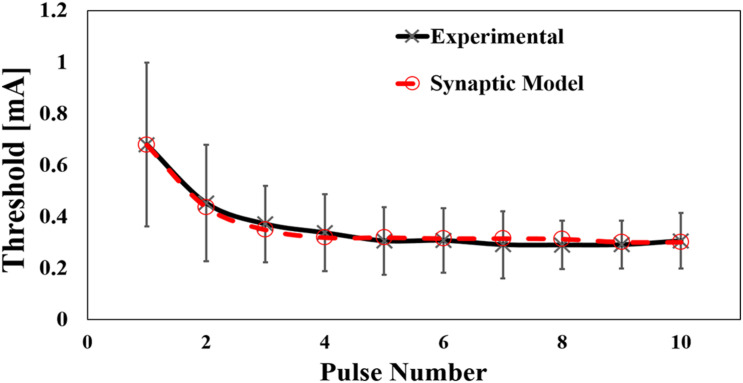
Computed results of the effect of pulse number by pulse-train electrical stimulation (experimental mean value and standard deviation, *n* = 8).

The computational results for the perception threshold are shown in Figure 6. The computed perception threshold corresponded to the injection current (IES) required to activate the postsynaptic neuron (that is, eliciting an action potential) using the integrated multiscale model of Aδ-fibers with the synaptic model with different numbers of stimulation pulses. A good match to the experimental results was obtained by changing the relatively small space parameter of the synaptic model. The mean error was 14 μA.

## Discussion

In this study, perception thresholds via selective stimulation of Aδ-fibers were measured using pulse-train stimulation. We hypothesized that a train of pulses can modify the perception threshold via synaptic effects. We observed threshold dependency on the number of pulses and the frequency variation. We then proposed a computational model for synaptic effects.

In the first experiment, we observed that an increase in the number of pulses decreased the threshold by approximately 2.7 times. This is attributed to a synaptic effect on the postsynaptic neurons in the ventral horn of the spinal cord as the number of afferent pulses is increased, and temporal summation explains the reduction in the measured threshold. We found that after a certain number of pulses (six pulses in our experiment), the threshold converged to a minimum. We considered that the postsynaptic neuron reaches the membrane potential threshold at this number of pulses, and additional pulses will not change the stimulation threshold. Moreover, we discarded any enhancement of the membrane potential of the Aδ-fibers at the terminal close to the needle electrode, considering the fixed frequency of the train-pulse stimulation (Reilly, 1989). In the second experiment, the frequency dependency of the perception threshold was investigated. We observed a U-shaped response with a minimum threshold of approximately 30 Hz and a reduction of 30% from the maximum value. The same phenomenon has been observed for phosphine perception thresholds during transcranial alternating current stimulation (Rohracher, 1935; Turi et al., 2013; Evans et al., 2019). Although the frequency at the minimum value was found to be between 16 Hz and 20 Hz, the difference may be due to the different characteristics of rod visual neurons. Based on the stimulation rate variation, we conjectured that facilitation (temporal/spatial summation) and the fatigue process of the synapse were the reasons for the U-shaped response (frequency variation experiment). The former reduced the stimulation threshold up to a certain stimulation frequency where a fatigue process (for example, depletion of vesicles that contain the neurotransmitter available at the synapses) initiated at higher rates (Simons-Weidenmaier et al., 2006; Ikeda and Bekkers, 2009). In both experiments we adopted normalization of the thresholds, showing a clear trend due to inter-variability factors (such as needle depth, tissue thickness, and Aδ-fiber distribution).

As an additional demonstration of the response of Aδ-fibers and synaptic effects, EEG recordings were conducted. We observed evoked responses at single pulse with detection time within a range of 0.33 s to 0.39 s (3 m/s) that agrees within conduction velocity of Aδ-fiber and is faster than that of C-fiber (0.5 m/s to 2 m/s) (Weiss et al., 2008), thus confirming the selectivity of Aδ-fiber. In addition, the synaptic effect was confirmed by recording the evoked potential dependency on the number of pulses. As an increasing number of pulses (up to five pulses) reduces the perception threshold, we hypothesized that the evoked potential disappears if the number of pulses is reduced while maintaining the injection current that generates a perception with six pulses. We found that two and four consecutive pulses did not produce an evoked response.

In our recent study, a multiscale model of Aδ-fibers was proposed and verified using S-D measurements for a single pulse condition to estimate perception thresholds (Tanaka et al., 2021). Based on this model, we incorporated a synaptic model to estimate the temporal effects of a train of pulses for the first time. Our multiscale model coupled with a synaptic model explained that a higher number of pulses of the stimulation waveform increased the number of descending pulses on each activated Aδ-fiber, thus facilitating the activation of postsynaptic neurons with fewer presynaptic fibers. Therefore, to activate fewer Aδ-fibers, a smaller injection current was required. A limitation of the proposed model is reproducing the results of the measured threshold under different frequencies. This requires extending the synaptic model to include potential fatigue processes at higher stimulation rates, which needs to be investigated in future work. Finally, the current methodology can be applied to other small fibers, such as C-fibers, in future studies.

## Data Availability Statement

The raw data supporting the conclusions of this article will be made available by the authors, without undue reservation.

## Ethics Statement

The studies involving human participants were reviewed and approved by Ethical Committee of the Nagoya Institute of Technology. The patients/participants provided their written informed consent to participate in this study.

## Author Contributions

TW and AH conceived and designed the study. TW and ST conducted the experiments. JG-T and ST conducted the simulation experiments. TW, ST, and JG-T processed the data. All authors analyzed the data, wrote the manuscript, and read and approved the manuscript.

## Conflict of Interest

The authors declare that the research was conducted in the absence of any commercial or financial relationships that could be construed as a potential conflict of interest.
