# Supplementary material for: Synaptic Effect of Aδ-Fibers by Pulse-Train Electrical Stimulation
Source: Front Neurosci. 2021 Apr 26;15:643448. doi: 10.3389/fnins.2021.643448 (PMC8107290; doi:10.3389/fnins.2021.643448)
Supplement: Supplementary file 1 [file Image_1.pdf]

Supplementary Material

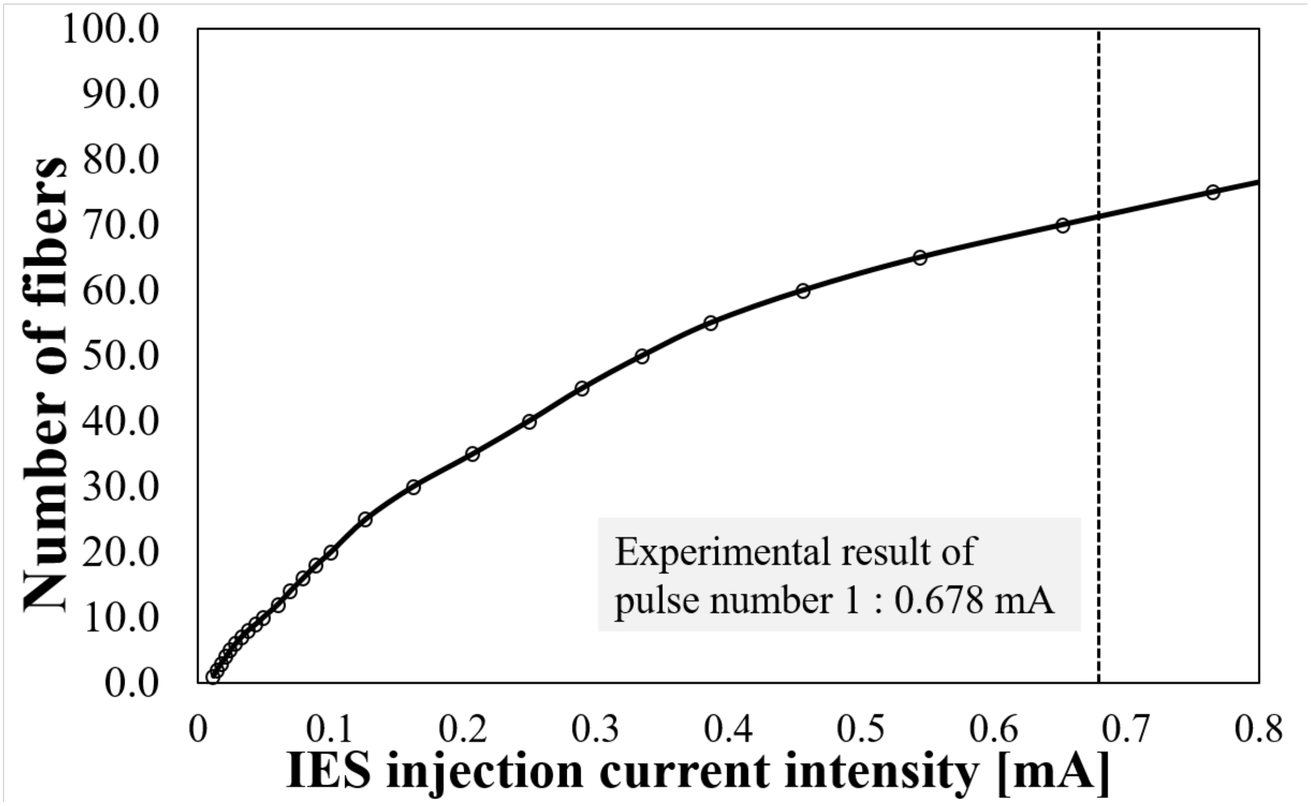

Supplementary Figure 1. The relationship between the input current and the number of nerves by using Aδ-fibre model.
